# How and When Job Crafting Relates to Employee Creativity: The Important Roles of Work Engagement and Perceived Work Group Status Diversity

**DOI:** 10.3390/ijerph18010291

**Published:** 2021-01-02

**Authors:** Wenqing Tian, Huatian Wang, Sonja Rispens

**Affiliations:** Department of Industrial Engineering & Innovation Sciences, Eindhoven University of Technology, 5612 AZ Eindhoven, The Netherlands; wendytwq@163.com (W.T.); s.rispens@tue.nl (S.R.)

**Keywords:** job crafting, work engagement, perceived work group member status diversity, creativity, diary study

## Abstract

Creative employees are treasured assets for organizations. However, relatively little is known about what specific actions employees can take to manage their own creative process. Taking a motivational perspective, this study examined how job crafting behaviors positively link to employee creative performance through work engagement, and whether perceived work group status diversity moderates this relationship. We conducted a weekly diary study in which 55 employees from a Chinese energy company were asked to fill in diaries over four consecutive weeks (176 observations in total). Results of the multilevel analyses showed that weekly job crafting behaviors were positively related to weekly creative performance through increasing weekly work engagement. In contrast to our expectation, we found that weekly job crafting behaviors were more positively related to weekly creative performance when perceived work group status diversity was high. In summary, our study suggests that job crafting behaviors are effective actions employees can take to manage their creative processes through increasing work engagement. In addition, we stress that status diversity in existing work environments is an important contextual factor that shapes the job crafting process.

## 1. Introduction

For gaining competitive and sustainable advantages in today’s dynamic business environment, creative employees are treasured assets for organizations [1,2]. To date, many studies have identified which personality traits and personal abilities could be associated with creative outcomes [3]. For example, research suggests that extrovert and flexible individuals tend to be more creative [4]. Employees with high growth needs and a learning orientation show higher levels of creative outcomes [5,6]. This literature also indicates that managers and organizations should build work environments that support employee creativity by setting creativity work goals, leading in a transformational manner, and rewarding employees when they achieve creative outcomes [5,7,8].

However, creativity is not only a matter of who is creative and how organizational contexts support employee creativity but also a matter of what actions employees themselves can take to enhance their creative process. It is surprising that relatively little is known about what specific actions employees actually take and how these actions contribute to creative performance. Although a limited number of studies identified some behavioral processes associated with creativity such as help/feedback-seeking behaviors [9,10], networking [11], and social learning [12], we recognize that these studies tended to take a resource- or information-based perspective. That is, these studies posit that individual help/feedback-seeking behaviors positively link to creativity through obtaining a broader base of resources and information [9,10]. However, few studies take a motivational perspective to understand the creative process. The question remains whether certain self-initiated actions and strategies may enhance creative performance. This might be an important omission as research suggests that actions and strategies driven by one’s intrinsic motivation are vital and beneficial to maintaining positive states at work and fostering work achievements [13,14].

Accordingly, this study proposes that job crafting, referring to the self-initiated changes employees make in their job demands and resources [15], may positively relate to employee creativity. The job crafting literature indicates that job crafting has significantly positive effects [1,16,17]. For example, job crafting is able to help employees to achieve goals, to take control, to find meaning in work, and to fulfil the need for connection [18]. Moreover, research shows that via job crafting, employees can experience positive emotions and reach an engaged state with vigor, dedication, and absorption at work [19,20]. Previous studies found that engaged employees are often intrinsically motivated to invest efforts to push work forward and come up with new ideas [21,22]. Therefore, we further propose that work engagement may be a motivational mediator transmitting job crafting behaviors into improved creative performance.

Nevertheless, the motivational process by which job crafting links to creativity may be contingent on certain conditions. In addition to personal conditions such as personality traits, capabilities, and/or personal resources [23], another important contextual factor is one’s work environment. The reason is that work environments consist of different colleagues and supervisors with whom employees regularly interact. This may enable or restrict the opportunities for employees to see what paths are available in how they craft their jobs [24]. Nowadays, work environments are more diverse than they used to be [25]. It is found that the working employees of contemporary organizations not only have heterogeneous knowledge, skills, and functional backgrounds but also present different levels of social status, power, and influence [12,26,27]. Although previous literature recognized that work group functional/informational diversity can facilitate information elaboration and offers individuals a resource-rich environment [28,29], surprisingly, few studies considered that work environments may also contain members’ social status differences, which may affect employees’ job crafting processes. For example, in a status-diverse work group, most decisions and regulations may be set up by authority figures [30,31]. With less autonomy, employees likely are less motivated to modify their job boundaries and activate thought–action processes [32]. Given its importance, this study incorporated the important role of perceived member status diversity in work environments.

To summarize, our study aimed to investigate how job crafting behaviors positively relate to employee creativity through work engagement and to what extent perceived member status diversity affects this positive relationship. We conducted a weekly diary study to address these questions. Using a diary method, this study was able to capture whether the weekly fluctuation in job crafting is related to weekly fluctuations in creativity and work engagement. Our study aimed to contribute to the literature in two ways. First, we contributed to the creativity literature by underscoring that job crafting is a potent action strategy that employees can use to manage their creative process and to achieve creative outcomes. Using a weekly diary design, we advanced the understanding of how job crafting links to employee creative performance through work engagement on a weekly basis. We uncovered how work engagement serves as a motivational mediator transmitting the benefits of job crafting into improved creative achievements. Second, we highlighted a boundary condition of the job crafting–creativity relationship. This study took a workplace diversity lens and uncovered an important contingent factor—perceived work group member status diversity—and how it may influence employees’ motivational processes. Our study thus provides a more nuanced insight into whether job crafting can be a successful strategy in a less favorable work environment.

## 2. Theory and Hypothesis Development

### 2.1. Job Crafting and Creativity

Creativity, in this study, refers to the generation of domain-specific, novel, and useful outcomes [6,8]. Therefore, we take creativity as a form of work outcomes at work, rather than a personality trait. We argue that job crafting strategy is positively related to employees’ creative outcomes. Job crafting is defined as a behavioral process by which employees redesign their jobs in order to fit their abilities and preferences, thus enhancing personal outcomes [24]. Building upon the job demands-resources model and job crafting theory [24], job crafting involves several proactive behaviors including seeking resources, seeking challenges, reducing demands, and optimizing demands [33,34,35]. Via job crafting, employees deliberately seek job resources that facilitate work outcomes and/or optimize those job demands that make them feel exhausted and stressful. Job crafting can not only enlarge the pool of cognitive resources but also stimulate the need for personal growth [18]. For example, seeking resources that accord with employees’ own needs can stimulate intrinsic as well as extrinsic motivation, which yields positive work outcomes [15,17,35]. Seeking challenges can foster mastery experiences, which in turn promote well-being and willingness to spend effort at work [17,35]. Optimizing demands, rather than simply reducing demands, leads to efficient work and a benefit with a secondary, self-serving gain (e.g., time) by using knowledge and skills to create and execute an alternative, more efficient path to that goal [33]. Therefore, it can be expected that job crafting motivates employees to invest efforts to push work forward and to come up with new ideas for improving work processes. Prior empirical studies and meta-analyses provide evidence of the link between job crafting and creativity [23,36]. For example, Demerouti and colleagues found that job crafting was positively related to employee creativity via increasing work engagement and flourishing [37]. Gordon and her team found that seeking resources was positively related to task performance and creativity [38]. Lin, Law, and Zhou found that job crafting was positively related to creativity and organizational citizenship behavior [39]. Taken together, we hypothesized that:

**Hypothesis** **1.** *Job crafting is positively related to creativity.*

### 2.2. Work Engagement as A Mediating Mechanism

Previous studies tended to take social network or information exchange perspectives (i.e., a sociological perspective) to explain the mechanism of resource-seeking behaviors (e.g., job crafting) on work-related outcomes [10,40]. However, the understanding of the job crafting process may not be complete. The job crafting process is not only rooted in how different information is processed but also in how employees are intrinsically motivated to enact their jobs. Therefore, this study used a motivational processing perspective (i.e., a psychological perspective) to underpin the relationship between job crafting and creativity.

The motivational processing perspective is framed on the job demands-resources model and self-determination theory [41,42,43]. That is, job crafting can be seen as a motivational process by which employees proactively adjust their job resources and demands, which increases the likelihood that the workplace satisfies one’s basic psychological needs (i.e., autonomy, competence, and relatedness) [44]. By being proactive, employees find motivating challenges and engage in effective problem solving, which enhances their engagement [45]. Moreover, the job demands-resources theory also posits that job resources play a key role in facilitating engagement because they can act as intrinsic or extrinsic motivators [46]. Hence, job crafting, as a bottom-up approach to mobilize resources, can be expected to facilitate work engagement. Empirical evidence showed that job crafting was positively related to work engagement and meaningful work [47], and daily job crafting is positively related to daily work engagement through momentary need satisfaction and momentary engagement [44,48]. Meta-analyses also confirmed that job crafting is a promising tool to stimulate work engagement [23,49].

Subsequently, work engagement can be expected to drive creativity. Work engagement refers to feelings of energy and enthusiasm about one’s work and consists of three dimensions: vigor, dedication, and absorption [20]. Based on a motivational processing perspective, engaged employees are intrinsically motivated to pursue their goals [19], are flexible in their thinking, and invest considerable effort in their work [21,22,50]. Work engagement provides employees with intrinsic task motivation, which is a necessary component for reaching creative solutions [51]. That is, those who are engaged will be motivated to use the skills and expertise needed to perform creatively [21]. Additionally, engaged employees often experience positive emotions, which widen their momentary thought–action repertoire process and generate personal resources [19]. These positive emotions facilitate creativity by fostering the thirst for exploring and assimilating new information [52]. When employees reach a high level of work engagement, positive work-related outcomes can subsequently emerge. For example, Bakker and Xanthopoulou in a study of a school principals and teachers dyad reported a mediating role of work engagement in the job resources–creativity relationship [21]. Similarly, Demerouti and colleagues found a positive link between work engagement and supervisor rated creativity in a study among employees of various sectors in the Netherlands [37]. Further, in a study among eldercare nurses in Japan, Toyama and Mauno demonstrated that work engagement mediated the relationship between emotional intelligence and creativity [53]. Taken together, we hypothesized that:

**Hypothesis** **2.** *Work engagement mediates the positive relationship between job crafting and creativity.*

### 2.3. The Moderating Role of Perceived Work Group Member Status Diversity

We further propose that it is also important to understand what conditions affect the relationship between job crafting and creativity. Research suggests that employees’ work environments form an important contextual factor that affects the relationship between job crafting and creativity [23,49]. Work environments have become more diverse than they used to be [25]. Besides knowledge/informational diversity in work environments, we recognize that work environments also include status diversity in which members may present different social status, power, and privilege [29,54,55,56]. The level of perceived status diversity in the work environment may have consequences for the motivational process by which job crafting links to creativity. Hence, it is important to consider the context of perceived work group member status diversity.

Status diversity in work environments tends to be seen as an unfavorable condition [54]. The literature on work group diversity indicates work group member status diversity tends to imply the vertical differences among group members concerning decision-making authority, power, and pay [54]. Research suggests that marked differences in group member status diminished group performance by distracting members from key tasks and interrupting the flow of information [54]. Based on the job demands-resources theory, work environments characterized by a higher level of member status diversity tend to restrict access to critical job resources. The reason may be that some crucial resources such as challenging opportunities, supervisory feedback, prior experience, and high-quality relational networks are controlled by a limited number of high-status members. As a result, lower-status members may have difficulty in accessing crucial resources and may have less motivation to access job resources that are controlled by higher-ranking members [57,58]. Hence, we argue that perceived job crafting opportunities become less available when working in a highly status-diverse work environment, impeding the motivational process.

Besides the fewer perceived opportunities for job crafting, employees likely have less autonomy to modify their job requirements and to seek new resources to facilitate their work processes when working in highly status-diverse work environments [27,29,55]. This is because most decisions and regulations may be set up authoritatively [30,31]. With less autonomy, employees would be less motivated to think creatively and to activate thought–action processes [32]. Hence, the relationship between job crafting and creativity may become weaker when employees perceive a highly status-diverse work environment. Prior studies and meta-analysis underscored the negative consequences of a status-diverse work environment such as job dissatisfaction, exhaustion, inequality, and resource-seeking barriers. Oedzes and his colleagues found that higher status-diverse work environment related negatively with team creativity when leaders exhibited little empowering behavior [59]. Similarly, Mullen, Johnson, and Salas demonstrated that individuals positioned at the lower levels of an informal hierarchy are often reluctant to share ideas and refrain from voicing their views in the presence of more influential authority figures [60]. Hence, we hypothesized that:

**Hypothesis** **3.** *Perceived work group member status diversity weakens the positive relationship between job crafting and creativity, such that this relationship becomes weaker when perceived work group member status diversity is high (vs. low).*

**Figure 1:**
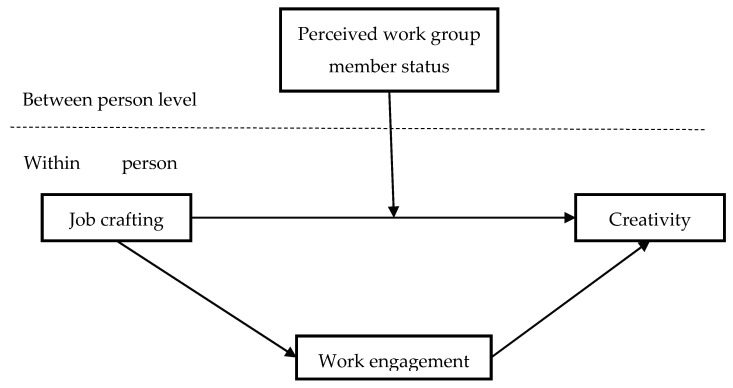
Conceptual model.

Therefore, based on our proposed hypotheses, we frame a conceptual model for visualization (see Figure 1).

## 3. Methods

### 3.1. Procedure and Participants

This study used a weekly diary study design. Although data collection can be practically difficult, research shows that diary design with repeated measures has more methodological advantages than cross-sectional and multi-wave designs [61,62,63]. For example, diary methods are a useful method of capturing the short-term dynamics of experiences, feelings, and behaviors within and between individuals in the work context. Diary studies provide researchers with the opportunity of capturing “life as it is lived” [64] (p. 597). Furthermore, diary studies are less susceptible to retrospective bias [65], which is known to threaten the validity of general survey measures.

In this study, participants were required to complete a survey questionnaire every week for four weeks and a general questionnaire at the start. Participants were employees of a medium-sized Chinese energy company. The second author informed the employees that the research was a weekly survey over four consecutive working weeks. Participants volunteered to fill in the anonymous questionnaire. The first author sent out the online questionnaire link to participants each Thursday and inspected the accomplishments by the end of every Friday. Each participant had a personal identification code, which enabled us to link each weekly entry. Finally, 44 out of 53 participants (i.e., 176 usable responses) were obtained, yielding an 83% response rate across weeks and individuals. We also conducted a power analysis to test whether 44 participants with repeated measures (i.e., 176 data points) had significant statistical power [66]. Results showed that repeated measures including examining within–between interactions in the equations should at least yield 36 sample size if statistical power is expected to be above 95%. This result was actually in line with the review article of Ohly and colleagues, indicating that sample size (person level) should be at least 30 to avoid biased estimates [61].

The final sample consisted of male (63.6%) and female (36.4%) participants. Their average age was 39.25 years, the standard deviation (SD) was 8.41, and average tenure was 13 years. A total of 41.5% of the respondents had a bachelor’s degree or above.

### 3.2. Measures

The questionnaires were in Chinese, and we conducted back-translation to ensure their validity. Unless otherwise stated, all measures used a 5-point Likert scale (1 = strongly disagree, 5 = strongly agree). Among them, we measured job crafting behaviors, work engagement, and creativity (i.e., within-person variables) over four consecutive weeks; while we only measured perceived work group member status diversity (i.e., the between-person variable) once in the general questionnaire together with demographic information. The questionnaires are shown in Appendix A.

Weekly job crafting was measured with 14 items [33,34], including 6 items for seeking resources (e.g., This week, I asked colleagues for advice. Cronbach’s alpha (α) ranged from 0.79 to 0.89 over four weeks), 3 items for seeking challenges following the scale validated by Petrou and colleagues [34] (e.g., This week, I asked for more responsibilities. α ranged from 0.79 to 0.93), and 5 items to measure optimizing demands using the scale of Demerouti and Peeters [33] (e.g., This week, I improved work processes/procedures to make my job easier. α ranged from 0.89 to 0.94).

Weekly creativity was measured with 4 items using the scale of Welbourne and colleagues [67]. An example item is “This week, I implemented new ideas during work”. The Cronbach’s alpha for each week ranged from 0.88 to 0.94.

Weekly work engagement was assessed with a 7-point Likert scale (1 = strongly disagree, 7 = strongly agree) [20]. We adapted the 3-item scale of Schaufeli and Bakker [20]. An example item is “This week, I felt bursting with energy.” The Cronbach’s alpha for each week ranged from 0.81 to 0.87.

Perceived work group member status diversity was defined as the vertical differences among group members concerning decision-making authority, power, and pay [54]. We used Harrison and Klein’s (2007) measure. An example item is “Are there significant differences in socioeconomic status and power among group members?” [54].

Finally, we measured gender and tenure as control variables due to their interference influences [68,69].

### 3.3. Analytical Approach

To examine the mediating effect, a bias-corrected bootstrapping analysis is recommended [70]. As our data include two levels, person-level and week-level, we used the MLMED macro to examine within-level mediation and between-level mediation [71]. To examine the moderating effects, we used the MLwiN program to conduct a multilevel regression. All week-level variables were centered on the person-mean avoiding multicollinearity and spurious regression. We first established a null model including only the intercept. In model 1, we entered the two control variables gender and tenure. In model 2, the main effects were entered, i.e., the predictors including specific dimensions of job crafting separately. In model 3, we entered the two-way interactions, i.e., predictors and the moderator (i.e., perceived work group member status diversity). Because our predictors were within-person variables but the moderator a between-person variable, we conducted a cross-level moderation analysis. In our multilevel regression, random effects of the slopes were examined. To test the improvement of each model over the previous one, the differences of its log-likelihood statistic −2×log and its chi-square (χ^2^) were computed.

## 4. Results

### 4.1. Preliminary Analysis

First, we conducted a multilevel confirmatory factor analysis (CFA) to analyze if the five indicators at the within-person level (i.e., weekly seeking job resources, weekly seeking job challenges, weekly optimizing job demands, weekly work engagement, and weekly creativity) were distinct constructs. Results of CFA with all five within-person level variables as separate constructs showed acceptable fit indices with χ^2^ = 110.602 (with degree of freedom (df) as 67), comparative fit index (CFI) as 0.955, Tucker–Lewis index (TLI) as 0.939, standardized root mean square residual (SRMR) as 0.045, and root mean square error of approximation (RMSEA) as 0.062. These results indicated that the constructs are sufficiently distinct from one another. Moreover, this model was significantly better than the model collapsing seeking resources, seeking challenges, and optimizing demands into one factor (χ^2^ = 336.075 (df = 74); CFI = 0.729; TLI = 0.667; SRMR = 0.126; RMSEA = 0.144; Δχ^2^ (7) = 225.473, *p* < 0.001).

Additionally, to justify the multi-level analysis, the intraclass correlation coefficient (ICC) was calculated, which examines the between-person and within-person variance components of the week-level constructs. The between-person variance of seeking resources, seeking challenges, optimizing demands, and creativity was 44.8%, 61.8%, 51.2%, and 35.8%. Thus, our variables varied both within and between persons, indicating that multilevel analysis was indeed appropriate.

Table 1 shows the descriptive statistics. Means, standard deviations, and correlations of each variable are summarized.

### 4.2. Hypothesis Testing

In testing H1, we found that weekly seeking challenges and weekly optimizing demands were positively related to weekly creativity (b = 0.201, *p* < 0.01; b = 0.234, *p* < 0.01) (see Model 2 in Tables 4 and 5). However, weekly seeking resources was not (b = −0.091, *p* > 0.05; b = −0.026, *p* > 0.05) (see Model 2 in Table 6). Hence, H1 was partially supported. The results indicated that weekly job crafting behaviors such as seeking challenges and optimizing demands were positively related to weekly creativity.

To test H2, we examined both within-person (i.e., week-level) mediation and between-person (i.e., person-level) mediation. For the week-level mediating effects of work engagement, we found b = −0.118, CI = [−0.204, −0.048] for seeking resources; b = 0.182, CI = [0.099, 0.279] for seeking challenges; and b = 0.175, CI = [0.092, 0.273] for optimizing demands (see Table 2). For the person-level mediating effects of work engagement, Table 3 shows that b = 0.201, CI = [0.049, 0.387] for seeking resources, b = 0.144, CI = [0.023, 0.293] for seeking challenges, and b = 0.189, CI = [0.055, 0.364] for optimizing demands. Results were statistically significant as the CI did not include zero. Thus, H2 was supported. Our results indicated that weekly job crafting behaviors were positively related to weekly creativity through increasing weekly work engagement. This mediating effect did not only exist within persons but also between persons.

**Figure 2:**
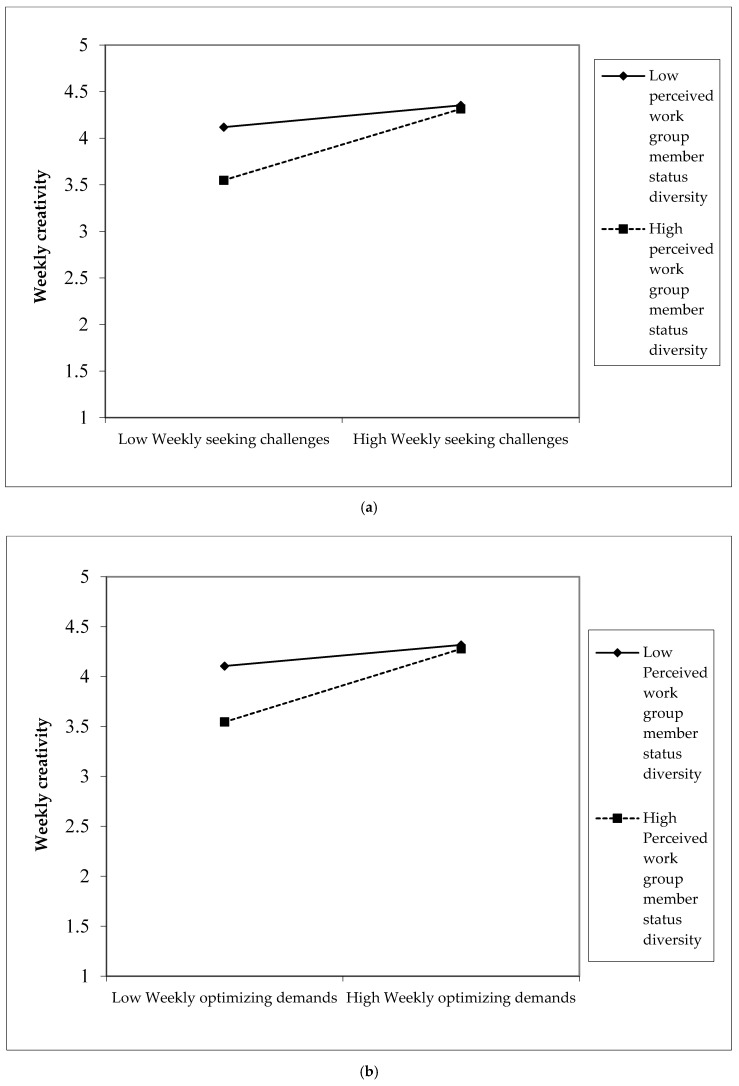
(**a**) The interaction of perceived work group status diversity and weekly seeking challenges on weekly creativity, (**b**) the interaction of perceived work group status diversity and weekly optimizing demands on weekly creativity.

Regarding H3, we found that the interaction between perceived work group member status diversity and weekly seeking challenges on weekly creativity was significant (b = 0.126; *p* < 0.05) (see Model 3 in Table 4). It was also significant for weekly optimizing demands (b = 0.143; *p* < 0.05) (see Table 5), but not significant for weekly seeking resources (b = −0.080; *p* > 0.05) (see Table 6). For the significant interactions between seeking challenges and optimizing demands approaches, we conducted simple slope analyses [72]. The simple slope analyses results showed that weekly seeking challenges and weekly optimizing demands were positively related to weekly creativity when perceived work group status diversity was high (b = 0.382, *p* < 0.05 for seeking challenges; b = 0.424; *p* < 0.05 for optimizing demands), while they were not significantly related to weekly creativity when perceived work group status diversity was low (b = 0.116, *p* > 0.05 for seeking challenges; b = 0.122; *p* > 0.05 for optimizing demands). As Figure 2a,b shows, weekly seeking challenges/optimizing demands was positively related to weekly creativity when perceived work group status was high. These results were in contrast with H3. Hence, H3 was not supported. Our results indicated that perceived status diversity strengthened the positive relationship between weekly job crafting behaviors and weekly creativity.

## 5. Discussion

In this study, we took a motivational processing perspective to advance the understanding of how and when job crafting behaviors positively relate to employee creativity. Our results showed that employees who engaged in weekly job crafting behaviors also reported more creative performance on a weekly basis. Moreover, weekly work engagement mediated this positive relationship. Surprisingly, we found that weekly job crafting behaviors were more positively related to weekly creative performance when perceived work group member status diversity was high. Taken together, our study suggests that job crafting is a potent way to sustain a higher level of creativity at work. Using a weekly diary design, we shed light on how job crafting is related to employee creative performance on a weekly basis. We stressed the mediating role of work engagement transmitting the benefits of job crafting into improved creative performance. We also added to the job crafting literature by uncovering an important contingency—perceived work group member status diversity. We provided an insight into a compensating effect of job crafting on creative outcomes when working in a status-diverse work environment.

### 5.1. Theoretical Contributions

This study mainly contributes to the literature in two ways. First of all, although prior studies uncovered factors affecting employees’ creativity, most of them have focused on identifying the personality characteristics and traits associated with creative outcomes [4,5,6]. This study contributes to the creativity literature by taking a behavioral lens to examine the means (i.e., job crafting) that can enhance employees’ creativity. Although researchers have proposed that various cognitive and behavioral processes occur in creativity [8], empirically, little is known about the specific strategies that employees use to manage their creative processes and how these strategies operate in the work context on a weekly basis. This study demonstrated that the job crafting strategies—seeking challenges and optimizing demands—were positively related to creativity. This study provides insights into how creativity can be improved by specific job redesign strategies.

This study established the mediating role of work engagement. Whereas prior studies took social network or information exchange perspectives to explain the mechanism of resource-seeking behaviors (e.g., job crafting) on work-related outcomes [10,40], we proposed a motivational perspective. Our results suggest that job crafting enables employees to become engaged in their work (i.e., vigor, dedication, and absorption), which, subsequently, facilitates thinking of novel solutions and performing creatively.

Second, this study further answers the question of under what conditions job crafting is more positively related to creativity. Although prior studies uncovered several important boundary conditions of job crafting such as personal traits and abilities, job autonomy, perceived organizational support, and organizational change context [35,73,74], this study took a workplace diversity lens to explore a contingency to job crafting. We examined perceived status diversity. Work group status diversity represents a vertical disparity in terms of decision-making authority, power, and pay [54]. A highly status-diverse work environment is often seen as an unfavorable work environment [57,58]. However, we found that weekly job crafting behaviors were more positively related to weekly creativity when perceived work group member status diversity was high, which was not in line with our hypothesis. Job crafting behaviors seem more beneficial to creative performance when employees work in an environment in which members’ social status is highly disparate and power is distributed across their ranks. We argue that this reverse result can be attributed to a compensating effect of job crafting [23]. Job crafting is a bottom-up job redesign process by which employees proactively adjust their job resources and demands in order to restore fit between themselves and environments [24,35,75,76]. Hence, when work environments are not optimal for individual employees, they can adapt via job crafting (i.e., by seeking challenges and/or optimizing demands). Prior research suggested that the positive association between job crafting and work attachment would be stronger when employees experience tough times at work [77], and that job crafting can act as a strategy of employees to respond to organizational change [35]. Our finding is in accordance with prior evidence supporting the role of job crafting in disadvantageous work conditions. Therefore, we contribute to the creativity and workplace diversity literature by uncovering the beneficial effect of job crafting on creativity when working in an unfavorable environment.

Notably, we also have an unexpected finding on the mediating effects of work engagement. We found that seeking resources was negatively related to work engagement, and further decreased creativity within weeks (i.e., on the week level) (see Table 2), but this relationship was positive on the person level. This finding implies that the seeking resources strategy is generally beneficial for employees’ creativity through increasing work engagement, but seeking resources might produce detrimental effects within a short period of time. We think that this could be a case when employees seek resources that are not relevant to work tasks, or when employees need more time and energy to understand and use the resources, and thus may be distracted from their work goals [34]. Hence, when employees seek such resources, they may become less engaged during the work and in turn decrease the level of creativity.

### 5.2. Practical Implications

Our study also has several practical implications for organizations and management practitioners. First, we suggest that job crafting as a bottom-up job redesign strategy including seeking resources, seeking challenges, and optimizing demands is an effective approach to enhancing employees’ work engagement and creativity. Moreover, job crafting behaviors play a more beneficial role when employees work in an unfavorable environment. Therefore, organizations and managers may want to empower employees to redesign their jobs and to adjust their job resources and demands. For example, emerging studies demonstrate that job crafting training and interventions are an effective tool to enhance employee job crafting behaviors [78,79].

Second, although our results showed that job crafting strategy is effective when employees work in a status-diverse work environment, the work group diversity literature still indicates that work environments characterized by hierarchies, status disparity, and authorities are not favorable for knowledge sharing, decision quality, collective performance, and individual well-being [54,57,58]. Hence, we suggest that organizations should attempt to create a relatively fair and inclusive work environment. Idea generation and implementation need favorable work environments [28,80].

### 5.3. Limitations and Future Research

Our study has potential limitations, which represent future research directions. First, all constructs in our study were self-reported, which may raise the question of whether common method bias may explain the results [17,78,81]. However, we used a diary study, and the literature indicates that common method bias is less likely to be a serious concern when interaction effects are observed [82] and when constructs are measured over time [83]. Second, although perceptions are important for understanding what people feel, think, and do, future research may include more objective indicators of proactive behaviors [84] and workplace diversity (e.g., a Gini index to measure work group status diversity) [85,86,87]. Third, we also hold concerns on the generalizability of our study. We collected data from an energy company in China, which represents a single industry and a single culture. Future studies are necessary to obtain confidence that our findings are generalizable to other industrial settings and other cultures. Last but not least, we suggest that future studies can look into team level creativity. Doing so can allow researchers to gain insights into how different employees interact and collaborate with one another and in turn enhance collective creative outcomes [88].

## 6. Conclusions

This study sheds light on how and under what conditions job crafting behaviors relate to employee creative performance. Using a weekly diary study design, our study uncovered how job crafting positively links to creativity through work engagement on a weekly basis. Our study highlights an important contingency—perceived member status diversity in work environments—that affects the relationship between job crafting and creativity. Our study indicates that job crafting is a potent action to manage the creative process and can be a useful compensating strategy when working in a status-diverse work environment (i.e., when work environments are less favorable). Our study suggests that it is important for researchers and practitioners to pay more attention to the beneficial role of job crafting on a weekly basis and the context of member status diversity in existing work environments.

## Figures and Tables

**Table 1:** Mean, SD, and within-level (below the diagonal) and between-level (above the diagonal) correlations of the study variables (N = 176).

Variable	Mean	S.D.	1	2	3	4	5	6	7	8
1.Work Engagement	4.648	1.247	1	0.589 **	0.600 **	0.595 **	0.522 **	−0.088	−0.107	−0.050
2.Creativity	3.608	0.765	0.529 **	1	0.563 **	0.671 **	0.572 **	−0.239 **	−0.294 **	−0.133
3.Seeking Resources	3.217	0.848	0.226 **	0.253 **	1	0.686 **	0.637 **	0.107	−0.052	−0.137
4.Seeking Challenges	2.947	1.003	0.573 **	0.483 **	0.243 **	1	0.481 **	−0.018	−0.268 **	−0.131
5.Optimizing Demands	3.697	0.865	0.509 **	0.429 **	0.199 **	0.534 **	1	0.100	−0.094	−0.206 **
6.Perceived work group member status diversity	3.590	1.054	−0.070	−0.175 *	0.078	−0.013	0.070	1	−0.065	−0.127
7.Gender	1.360	0.482	−0.085	−0.215 **	−0.038	−0.196 **	−0.065	−0.065	1	0.124
8.Tenure	12.800	10.005	−0.039	−0.097	−0.100	−0.096	−0.143	−0.127	0.124	1

**. Correlation is significant at the 0.01 level (2-tailed); *. Correlation is significant at the 0.05 level (2-tailed).

**Table 2:** Results of the indirect effects of job crafting approaches on creativity through work engagement (within-level).

Variables → Mediator → Outcomes	Effect	SE	z	*p*	MCLL	MCUL
Seeking resources → work engagement → creativity	−0.118	0.041	−2.911	0.004	−0.204	−0.048
Seeking challenges → work engagement → creativity	0.182	0.046	3.962	0.000	0.099	0.279
Optimizing demands → work engagement → creativity	0.175	0.046	3.777	0.000	0.092	0.273

N = 176; unstandardized regression coefficients are reported; bootstrap sample size = 5000 bias corrected; LL = lower limit, UL = upper limit; significance level of confidence is at 95%.

**Table 3:** Results of the indirect effects of job crafting approaches on creativity through work engagement (between-level).

Variables → Mediator → Outcomes	Effect	SE	z	*p*	MCLL	MCUL
Seeking resources → work engagement → creativity	0.201	0.087	2.318	0.021	0.049	0.387
Seeking challenges → work engagement → creativity	0.144	0.069	2.097	0.036	0.023	0.293
Optimizing demands → work engagement → creativity	0.189	0.080	2.376	0.018	0.055	0.364

N = 176; unstandardized regression coefficients are reported; bootstrap sample size = 5000 bias corrected; LL = lower limit, UL = upper limit; significance level of confidence is at 95%.

**Table 4:** Multilevel estimates of weekly seeking challenges, perceived work group member status diversity on weekly creativity.

	Model 1			Model 2			Model 3		
	Estimate	SE	Sign	Estimate	SE	Sign	Estimate	SE	Sign
Constant	4.124	0.252	**	4.124	0.252	**	4.083	0.24	**
*Control only*									
Gender	−0.327	0.168		−0.327	0.168		−0.285	0.159	
Tenure	−0.005	0.008		−0.005	0.008		−0.007	0.008	
*Subjects*									
Seeking challenges				0.201	0.064	**	0.249	0.074	**
Status diversity							−0.144	0.074	
*Interactions*									
Seeking challenges x status diversity							0.126	0.061	*
-2LL	369.432			359.871			346.915		
d.f.	2			1			2		
-2LL differences	4.426			9.561	*		12.956	*	
Individual level variance	0.193	0.061	**	0.199	0.061	**	0.187	0.056	**
Week level variance	0.358	0.044	**	0.333	0.041	**	0.292	0.040	**

**. Correlation is significant at the 0.01 level (2-tailed); *. Correlation is significant at the 0.05 level (2-tailed); N = 176 data points; “status diversity” refers to “perceived work group member status diversity”; “-2LL” refers to -2*loglikelihood.

**Table 5:** Multilevel estimates of weekly optimizing demands, perceived work group member status diversity on weekly creativity.

	Model 1			Model 2			Model 3		
	Estimate	SE	Sign	Estimate	SE	Sign	Estimate	SE	Sign
Constant	4.124	0.252	**	4.124	0.252	**	4.062	0.239	**
*Control only*									
Gender	−0.327	0.168		−0.327	0.168		−0.278	0.159	
Tenure	−0.005	0.008		−0.005	0.008		−0.006	0.008	
*Subjects*									
Optimizing demands				0.234	0.070	**	0.273	0.076	**
Status diversity							−0.142	0.074	
*Interactions*									
Optimizing demands x status diversity							0.143	0.059	*
-2LL	369.432			358.664			343.321		
d.f.	2			1			2		
-2LL differences	4.426			10.768	*		15.343	**	
Individual level variance	0.193	0.061	**	0.200	0.061	**	0.187	0.056	**
Week level variance	0.358	0.044	**	0.330	0.041	**	0.295	0.039	**

**. Correlation is significant at the 0.01 level (2-tailed); *. Correlation is significant at the 0.05 level (2-tailed); N = 176 data points; “status diversity” refers to “perceived work group member status diversity”; “-2LL” refers to -2*loglikelihood.

**Table 6:** Multilevel estimates of weekly seeking resources, perceived work group member status diversity on weekly creativity.

	Model 1			Model 2			Model 3		
	Estimate	SE	Sign	Estimate	SE	Sign	Estimate	SE	Sign
Constant	4.124	0.252	**	4.124	0.252	**	4.170	0.243	**
*Control only*									
Gender	−0.327	0.168		−0.327	0.168		−0.344	0.161	*
Tenure	−0.005	0.008		−0.005	0.008		−0.007	0.008	
*Subjects*									
Seeking resources				−0.091	0.077		−0.136	0.084	
Status diversity							−0.146	0.074	*
*Interactions*									
Seeking resources x status diversity							−0.080	0.059	
-2LL	369.432			368.057			362.462		
d.f.	2			1			2		
-2LL differences	4.426			1.375			5.595		
Individual level variance	0.193	0.061	**	0.194	0.061	*	0.172	0.056	**
Week level variance	0.358	0.044	**	0.355	0.044	**	0.350	0.043	**

**. Correlation is significant at the 0.01 level (2-tailed); *. Correlation is significant at the 0.05 level (2-tailed); N = 176 data points; “status diversity” refers to “perceived work group member status diversity”; “-2LL” refers to -2*loglikelihood.

## Data Availability

The data are not publicly available due to privacy and ethical considerations.

## Appendix A

**General Information**

The questions in this section of the questionnaire concern your personal situation. Please write down your answer on the dotted line or check the box that represents the answer of your choice.

Q01	What is your age?	___years
Q02	What is your gender?	0 = Female 1 = Male
Q03	What is your highest education qualification?	1 = Vocational Training 2 = HO 3 = Bachelor’s 4 = Master’s 5 = PhD 6 = Other
Q04	What is your tenure in this company?	_______years
Q05	To what extent do you agree there are significant differences in socioeconomic status among group members?	1 = strongly disagree 2 = disagree 3 = neither disagree nor agree 4 = agree 5 = strongly agree

Weekly Questionnaire

Job Crafting						
In the following, we ask for your opinion about some questions of job crafting. Please circle the answer of your choice.						
Question number	To what extent do you agree or disagree with the following statements?	Never	Sometimes			Often
Week-level seeking resources						
Q01	I ask others for feedback on my job performance.	1	2	3	4	5
Q02	I ask colleagues for advice.	1	2	3	4	5
Q03	I ask my supervisor for advice.	1	2	3	4	5
Q04	I try to learn new things at work.	1	2	3	4	5
Q05	I contacted other people from work (e.g., colleagues, supervisors) to get the necessary information for completing my tasks.	1	2	3	4	5
Q06	When I have difficulties or problems at my work, I discuss them with people from my work environment.	1	2	3	4	5
Week-level seeking challenges						
Q07	I ask for more tasks if I finish my work.	1	2	3	4	5
Q08	I ask for more responsibilities.	1	2	3	4	5
Q09	I ask for more odd jobs.	1	2	3	4	5
Week-level optimizing demands						
Q10	I simplified work processes/procedures to make my job easier.	1	2	3	4	5
Q11	I thought of solutions in order to carry out work more easily.	1	2	3	4	5
Q12	I improved work processes/procedures to make my job easier.	1	2	3	4	5
Q13	I looked for ways to make my work more efficient.	1	2	3	4	5
Q14	I tried to change them when certain work processes/procedures slowed down.	1	2	3	4	5

	Creativity/Innovation					
Considering all your job duties and responsibilities, how would your supervisor or manager rate your behaviors at work this week? Please circle the answer of your choice.						
**Question number**	**This Week, I…**	**Strongly Disagree**		**Agree**	**Strongly Agree**	
Q15	Came up with new ideas	1	2	3	4	5
Q16	Worked to implement new ideas	1	2	3	4	5
Q17	Found improved ways to do things	1	2	3	4	5
Q18	Created better processes and routines	1	2	3	4	5

Work Engagement								
Think of this week; to what extent do you agree with the following statements?								
**Question number**	**This Week…**	**Strongly Disagree**	**Agree**					**Strongly Agree**
Week-Level Vigor								
Q19	I felt bursting with energy.	1	2	3	4	5	6	7
Q20	My job inspired me.	1	2	3	4	5	6	7
Q21	I got carried away when I was working.	1	2	3	4	5	6	7
